# Editorial: Dyslipidemia, prevention in the era of personalized medicine?

**DOI:** 10.3389/fmed.2025.1756918

**Published:** 2026-02-11

**Authors:** Amani Kallel, Maciej Banach

**Affiliations:** 1 Tunis El Manar University, Tunis, Tunisia; 2 Faculty of Medicine, The John Paul II Catholic University of Lublin, Lublin, Poland

**Keywords:** apolipoprotein B, atherosclerotic cardiovascular disease, bempedoic acid, dyslipidemia, evidence-based medicine, proprotein convertase subtilisin/kexin type 9 (PCSK9), personalized medicine, sex/gender differences

## Introduction

Cardiovascular disease (CVD) remains the leading cause of mortality worldwide, and dyslipidemia stands as one of its most significant modifiable risk factors. As we advance into an era of personalized medicine, a fundamental question emerges: can we truly prevent dyslipidemia through individualized approaches that account for each patient's unique genetic, physiological, and environmental context? This Research Topic brings together compelling evidence from diverse patient populations, challenging us to reconsider traditional prevention strategies and embrace more nuanced, patient-centered approaches.

The manuscripts assembled here examine dyslipidemia across hypertensive populations, hemodialysis patients, individuals with diabetes and metabolic dysfunction-associated steatotic liver disease (MASLD), and post-menopausal women—each representing unique prevention challenges and opportunities that illuminate the path toward truly personalized medicine.

## Understanding population-specific risks

Prevention begins with accurate risk identification. Wang et al. provide crucial epidemiological data through their cross-sectional study of 92,443 hypertensive patients, revealing an overall dyslipidemia prevalence of 37.5%. Importantly, they identified sex-specific patterns: triglyceride abnormalities were more common among males (16.8%), whereas total cholesterol abnormalities predominated in females (14.4%). Multiple factors associated with dyslipidemia prevalence included gender, age, diabetes, coronary heart disease, body mass index, central obesity, physical activity frequency, smoking status, and alcohol consumption—providing a foundation for developing personalized risk prediction models that could enhance primary prevention efforts in community settings.

## When interventions don't work: the importance of evidence

Personalized prevention must be grounded in evidence rather than theoretical assumptions. Karimi et al.'s systematic review and meta-analysis of 28 randomized controlled trials involving 1,340 hemodialysis patients revealed that L-carnitine supplementation does not significantly improve serum lipid profiles. Despite the biological plausibility of supplementation in this population experiencing carnitine deficiency, the meta-analysis demonstrates no benefit—a sobering reminder that personalized prevention strategies must be validated through rigorous clinical trials rather than extrapolated from mechanistic reasoning alone.

## Sex-specific biology and treatment responses

Perhaps nowhere is the need for personalized prevention more evident than in the recognition of sex-specific differences. Ábel et al. review extensive evidence showing that women with type 2 diabetes have a higher risk of atherosclerotic cardiovascular disease than men, though the exact mechanisms remain incompletely understood. The association between MASLD and type 2 diabetes appears bidirectional, with dyslipidemia common in both conditions, emphasizing that prevention efforts must address the entire metabolic syndrome constellation.

Regarding treatment efficacy, real-world data reveal concerning disparities. Statin therapy appears under-prescribed for both type 2 diabetics and patients with MASLD, with some studies showing lower rates in women compared to men. For newer therapies, results from real-world studies suggest that up-titration of statin dose improves the efficacy of PCSK9 inhibitors in women, indicating that personalized prevention may require optimization of foundational therapies before advancing to more intensive interventions, with treatment algorithms potentially differing by sex. Encouragingly, bempedoic acid has shown greater effectiveness in lipid lowering in women compared to men, suggesting that some novel agents may offer sex-specific advantages.

## Novel biomarkers for risk stratification

The potential of novel biomarkers to enhance personalized prevention is exemplified by Rottura et al., who investigated PCSK9 levels in 135 post-menopausal diabetic women in primary prevention. Apolipoprotein B values were an independent predictor of PCSK9 levels, while LDL values were inversely related. Critically, PCSK9 levels directly influenced pulse wave velocity, a validated marker of arterial stiffness and cardiovascular risk, suggesting that PCSK9 measurement could enhance risk stratification in post-menopausal diabetic women, potentially identifying individuals who would benefit from more aggressive prevention interventions before clinical cardiovascular disease develops.

## Principles for personalized prevention

The manuscripts collectively point toward several key principles:

Risk Stratification Must Be Multidimensional: Comprehensive risk assessment should incorporate body composition, comorbidities, family history, lifestyle factors, and potentially novel biomarkers such as PCSK9 and apolipoprotein B beyond traditional risk calculators.

Prevention Strategies Must Account for Sex: Men and women experience dyslipidemia differently, respond differently to treatments, and face different barriers to care. Prevention programs must be designed with these differences in mind, from screening protocols to treatment algorithms.

Comorbidity Patterns Define Unique Prevention Needs: Patients with diabetes and MASLD, those with hypertension, individuals with chronic kidney disease—each represents a distinct prevention scenario requiring tailored approaches.

Evidence Must Trump Biological Plausibility: The L-carnitine example reminds us that even theoretically sound interventions require validation. Personalized prevention should represent the application of high-quality evidence to individual patient contexts, not personalized experimentation.

## Conclusion

The question “Dyslipidemia, Prevention in the Era of Personalized Medicine?” is to be answered in a qualified yes. Indeed, personalized approaches are both necessary and feasible for dyslipidemia prevention, but their very implementation requires strong epidemiological data on population-specific risks, rigorous validation of clinical interventions, acknowledgment and correction of inequities in healthcare delivery, and the integration of traditional and newer biomarkers into risk assessment tools.

The manuscripts assembled here provide important evidence to support personalized prevention strategies and point to remaining challenges. Importantly, personalized prevention will increasingly depend on e-health tools, including digital risk calculators, mobile health applications for lifestyle modification monitoring, telemedicine platforms for remote monitoring, and artificial intelligence algorithms that permit the personalization of risk stratification, to scale personalized care. As we continue to unravel the complex interactions between genetics, sex hormones, metabolic diseases, and environmental factors driving dyslipidemia, the promise of precision prevention will increasingly become clinical reality through continued collaboration to translate these insights into practical prevention strategies that reduce the global burden of cardiovascular disease.

